# Untreated Psychosis and Healthcare‐Seeking Behaviour in Pakistan: A Qualitative Study With Young People, Carers, Traditional Healers, and Healthcare Professionals

**DOI:** 10.1111/eip.70233

**Published:** 2026-08-04

**Authors:** Noor Sanauddin, Lisa Dikomitis, Abdul Jalil Khan, Mian Mukhtar ul Haq Azeemi, Muhammad Firaz Khan, Saima Sheikh, Nishani Fonseka, Abbie Milner, Syed Muhammad Uzair Shah, Ishfaq Tariq Azeemi, Shumaila Hamid, Syeda Fatima Jamal, Aimee Sargeant, Saeed Farooq

**Affiliations:** ^1^ Department of Sociology University of Peshawar Peshawar Pakistan; ^2^ Warwick Medical School, University of Warwick Coventry UK; ^3^ Institute of Public Health & Social Sciences, Khyber Medical University Peshawar Pakistan; ^4^ Lady Reading Hospital Peshawar Pakistan; ^5^ Institute of Mental Health and Behavioural Sciences, Khyber Medical University Peshawar Pakistan; ^6^ Nuffield Department of Orthopaedics, Rheumatology and Musculoskeletal Sciences University of Oxford Oxford UK; ^7^ School of Medicine, Keele University Newcastle‐under‐Lyme UK; ^8^ University Hospitals of North Midlands NHS Trust Stoke‐on‐Trent UK

**Keywords:** ethnography, mental health, Pakistan, psychosis, stigma, traditional and spiritual healers

## Abstract

**Background:**

Prolonged delay between the onset of the first episode of psychosis (FEP) and initiation of treatment is common in lower‐ and middle‐income countries (LMICs), including Pakistan. This qualitative study explored perspectives of young persons with FEP, carers, healthcare professionals, and traditional and spiritual healers (TSHs) on drivers of delayed diagnosis and treatment of FEP, to suggest interventions aimed at addressing factors contributing to prolonged duration of untreated psychosis (DUP) in Pakistan and similar contexts.

**Methods:**

We conducted a qualitative study in Peshawar, Pakistan using focused ethnography as a research design. We first conducted focused ethnography using participant observation with 15 TSHs. Subsequently, data was collected through semi‐structured interviews (*n* = 26) and focus group discussions (*n* = 6) with FEP individuals (age 14–25), their carers, and healthcare professionals. We used framework analysis, combining inductive and deductive thematic approaches to analyse the data.

**Results:**

Three themes emerged from the analysis in relation to care pathways in psychosis: (1) moral‐spiritual explanations legitimise spiritual healings; (2) stigma renders psychiatric disclosure socially risky; and (3) structural exclusion of inaccessibility of mental health from primary care.

**Conclusion:**

Psychosis remains untreated for a prolonged time as families navigate a moral‐spiritual landscape that makes TSHs the only legitimate healers, stigma makes psychiatric disclosure a threat to family honour, and absent or dismissive services make biomedical care unreachable. Engagement with TSHs represents an underutilised opportunity to develop collaborative care models that reduce the duration of untreated psychosis.

## 1 Introduction

The first episode of psychosis (FEP) is a critical therapeutic time point. Good recovery outcomes can be achieved if patients are treated promptly and effectively (Farooq et al. 2009, 2024). However, individuals suffering from FEP often experience a significant delay between the onset of psychotic symptoms and the initiation of treatment. This is especially critical in low‐ and middle‐income countries (LMICs), where it is estimated that over 80% of all FEP takes place (Singh and Javed 2020). Significantly, two‐thirds of the population of Pakistan is below the age of 30, making it among the world's youngest nations (Ahmad 2018). Yet, early intervention in psychosis (EIP) services in Pakistan, like other LMICs, are underdeveloped and lacking. There is a severe shortage of mental health professionals, with recent estimates of around 500 registered psychiatrists for the entire population of over 245 million people (Sikander 2020).

The duration of untreated psychosis (DUP), defined as the time from manifestation of the first psychotic symptom to initiation of adequate treatment from a qualified mental health professional (Marshall et al. 2005), is likely to vary considerably in patients depending on their help‐seeking pattern. Measuring the DUP is a critical component in early intervention research. Because the onset of psychosis is typically a gradual process rather than an abrupt event, researchers utilise a variety of specialised assessment tools to retrospectively map this transition. The Nottingham Onset Schedule (NOS), developed by Singh et al. (2005), stands out as a highly reliable and validated instrument for measuring DUP.

Notably, DUP is alarmingly protracted in most LMICs, where the average DUP is 125 weeks, which is twice as long as the 63 weeks of DUP in high‐income countries (HICs) (Large et al. 2008). Lilford et al. found that DUP in LMICs ranges from a mean of 30–225 weeks (Lilford et al. 2020). Research demonstrates that a longer DUP is associated with a longer time to symptom remission, a lesser degree of recovery, a greater likelihood of relapse and a worse overall outcome (Farooq et al. 2009; Singh et al. 2005). Significant resources have been devoted to EIP on the grounds that reducing DUP improves health outcomes (Csillag et al. 2015).

Health‐seeking behaviour and pathways for mental illness are pluralistic and complex in Pakistan. Cultural variations in beliefs about psychosis and the structure of healthcare systems play a significant role in prolonging DUP (Burns and Tomita 2014). The three most frequently reported barriers to timely treatment include a preference for traditional and alternative treatments (33.33%), stigma (25%), and a low level of mental health literacy among people (25%) (Saade et al. 2023). Across many LMICs, traditional and spiritual healers (TSHs) constitute an important care pathway for people with mental illness (Farooq et al. 2024; Subudhi et al. 2017). Despite a large number of research studies, there is a limited understanding of how individuals and families navigate care after the onset of psychosis (Oliver et al. 2018). Greater insight into care pathways is needed to develop approaches that support timely engagement with appropriate care.

In Peshawar specifically, work by Farooq et al. highlighted long duration of untreated psychosis and limited psychiatric infrastructure (Farooq et al. 2023). Recent quantitative work in Peshawar linked younger age, low education, and sociocultural barriers to severe first‐episode psychosis (FEP) presentations, recommending collaboration with faith healers for early referral (Mohammad et al. 2026). A qualitative study in Peshawar identified structural barriers to schizophrenia care, including reliance on spiritual healing and absent referral pathways at basic health units (Khattak et al. 2022). Research to date emphasises patient and family perspectives or quantifies delays, but rarely engages traditional spiritual healers to understand their role in DUP and help‐seeking. Moreover, how structural constraints within Peshawar's formal health system shape pathways also remains largely under examined. To the best of our knowledge, no ethnography has been conducted in Pakistan to explore help‐seeking in FEP. This paper presents the results of a formative study in a larger research project called THEHOPE (*T*raditional *HE*alers working with primary care and mental *H*ealth for early interventi*O*n in *P*sychosis in young p*E*rsons), a randomised controlled cluster feasibility trial for early identification, referral and management of FEP in the young population in Peshawar, Pakistan (Farooq et al. 2023). This paper extends regional literature by exploring the socio‐cultural, religious, and health system‐related factors that influence help‐seeking behaviour for psychosis.

Moreover, few studies in Pakistan have used an explicit conceptual framework to examine help‐seeking sequences. This study applies Rogler & Cortes's ‘Pathways to Care’ framework (Rogler and Cortes 1993), which conceptualises help‐seeking as a socially negotiated sequence of contacts initiated by individuals and their networks. Accordingly, we aimed to explore pathways to care for psychosis by examining the roles of TSHs, families, and biomedical providers.

## 2 Methodology

### 2.1 Study Design

A qualitative, ethnographic research design was deemed most suitable for the current study, as the design provides a deeper understanding of experiences, phenomena and context (Cleland 2017). We used focused ethnography through participant observation among TSHs. Focused ethnography has emerged as a promising method for applying ethnography to a distinct issue in a shorter time and a specific setting (Knoblauch 2005; Cruz and Higginbottom 2013). While traditional ethnography relies on long‐term immersion to comprehensively understand a community's holistic culture and habitus (Madden 2017), focused ethnography employs a narrowed analytical lens to target specific variables, phenomena, or sub‐cultures within a compressed timeframe (Handwerker 2001). We used focused ethnography to enable a rapid yet in‐depth exploration of traditional and spiritual healing for psychosis. This approach provided a targeted scope and efficient data collection (Trundle and Phillips 2023), allowing us to generate culturally grounded insights into health‐seeking behaviours and treatment pathways for first‐episode psychosis specifically, and mental health conditions more broadly. To our knowledge, this approach has not been used previously in Pakistan in the context of psychosis. In addition, we also collected data through semi‐structured interviews (SSIs) and focus group discussions (FGDs) from young persons with psychosis, carers, psychiatrists, primary care physicians (PCPs) and general community members.

### 2.2 Study Setting

The study was conducted in Peshawar, the capital of Khyber Pakhtunkhwa province in Pakistan. With a population of over 4 million, Peshawar district is mostly populated with Pashtun ethnic groups, along with a significant number of Hindko speakers and a high population of Afghan refugees due to its proximity to Afghanistan. Participant observations were conducted in places where the TSHs practiced their treatment. Data from young persons with psychosis and carers was collected at the psychiatry ward of Lady Reading Hospital, while data collection from psychiatrists, primary care physicians, and the general community took place at Khyber Medical University.

Peshawar is characterised by a pluralistic health system where biomedical, traditional, and spiritual healing co‐exist, along with strong family involvement in care decisions. Mental health services are provided mainly in three teaching hospitals in Peshawar with about 20 qualified psychiatrists. While findings of this study are context‐bound, they may have theoretical transferability to other similar settings with similar structural features: limited mental health specialists, high reliance on informal providers, and cultural explanatory models attributing mental illness to spiritual causes.

### 2.3 Sampling and Sample Size

We conducted focused ethnography through participant observation among 15 TSHs, using purposive maximum variation sampling to recruit TSHs, reflecting diversity in healing tradition, age, gender, popularity, and geographic location across five different *Tahsils* (administrative divisions) of district Peshawar. Subsequently, we collected data through SSIs with young people (aged 14–25) who were diagnosed with FEP (*n* = 12) and their carers (*n* = 14). In addition, we conducted FGDs with PCPs (*n* = 7), psychiatrists (*n* = 5), TSHs (*n* = 7), general community members (*n* = 7), male carers (*n* = 6), and female carers (*n* = 4). Altogether, the sample size for this study was 77 participants, mostly selected through purposive and snowballing techniques. Data collection was guided by a reflexive and iterative approach. The core team conducted regular meetings with field researchers to discuss and resolve methodological and logistical challenges, discuss the transcription of data which was going on concurrently with data collection, examine emerging themes, and to consider the adequacy of the dataset. These ongoing analytic conversations helped us critically review our sampling strategy and decide on sample size. Consensus was reached that additional data collection was unlikely to yield new conceptual insights, indicating that thematic saturation had been achieved. Data collection was concluded at this point in line with principles of qualitative rigour.

### 2.4 Recruitment

A variety of strategies were used to recruit participants for this study. TSHs for ethnography were identified and recruited after a long process of mapping TSHs and community engagement activities in the district of Peshawar. We spent 3 months mapping TSHs in Peshawar, leading to the identification of 355 TSHs in the district. This registry of TSHs is the first of its kind in Pakistan. This was followed by the recruitment of 15 TSHs for participant observation based on their reputation for treating mental illnesses and their willingness to participate in the study. Given the sensitivities surrounding spiritual healing, establishing trust with TSHs required sustained engagement. Many TSHs were initially reluctant, reporting no prior research involvement and expressing concerns that the team might be government agents. Specific concerns included fear of criticism from biomedical professionals and misrepresentation of spiritual practices. We addressed these by emphasising the study's focus on documenting help‐seeking pathways rather than evaluating TSH effectiveness, confirming that participation was voluntary and non‐judgmental. Recruitment proceeded only after trust was established and TSHs expressed willingness to participate. Young persons diagnosed with FEP who were recently admitted to the psychiatry ward of Lady Reading Hospital, Peshawar, and their carers were recruited for interviews. PCPs working in the primary health care centres and psychiatrists in district Peshawar were recruited based on professional acquaintances of the research team.

We acknowledge that the hospital‐based strategy of recruitment introduces selection bias by excluding individuals who have not accessed biomedical services. This approach was necessitated by ethical and feasibility constraints as recruitment of individuals with untreated psychosis in community settings raises significant risks and ethical concerns regarding capacity, safeguarding, and distress. To mitigate bias, we included caregivers to provide retrospective accounts of help‐seeking prior to hospital contact and used maximum variation sampling to include participants with diverse pathways, including prior TSH consultation. We recruited TSHs from community settings independent of hospital referrals, which began via professional acquaintances to establish initial trust, given sensitivities described above. To reduce homogeneity, subsequent TSHs participants were identified through community leaders and snowball sampling from non‐acquaintance sources.

### 2.5 Data Collection

Data collection occurred over 6 months, from September 2022 to March 2023. We conducted focused ethnography involving sustained participant observation with selected TSHs. We visited each TSH 2–3 times to assess their suitability for ethnography and to obtain informed consent/approval for observations. These repeated visits enabled rapport‐building. Given constraints on TSHs’ time and sensitivities around observation of healing rituals, we prioritised depth of interaction in bounded settings over prolonged presence. The researchers collected data involving 80 h of direct participant observation in TSHs’ consultation spaces. The methodology specifically prioritised targeted, condensed field visits over long‐term immersion, with observation periods averaging 5 h per healer, excluding preliminary logistical efforts like site mapping, travelling, and time spent on rapport building and consent negotiations. There is no consensus among scholars regarding the amount of time in the field required for focused ethnography to be considered ‘real’ ethnography (Kelly 2022). A systematic review of focused and other forms of rapid ethnographies found that these methodologies engage in fieldwork ranging from 5 days to 6 months (Vindrola‐Padros and Vindrola‐Padros 2018). Handwerker (Handwerker 2001) has recommended that a quick ethnography can be conducted over a period of 3–90 days. Similarly, Isaacs (Isaacs 2013) spent one day visiting each site for her multi‐site research project. This suggests that rapid forms of ethnography measure fieldwork duration in hours and days rather than months or years (Cefkin 2013).

The observation element of focused ethnography adds a vital dimension as it can provide insights into subtle and hidden practices that respondents may not recall in interview settings (Rashid et al. 2019). Experts recommend the incorporation of other methods of data collection, such as in‐depth interviews, as an important vehicle for quickly providing a targeted wealth of information on the research topic (Kelly 2022).

A structured observation framework was prepared to help the ethnographers keep a focus on collecting and recording observations on important aspects of the healing practices and procedures, including the setting, the healers, the patients, the carers/families, interaction between healers and patients/carers, and researcher reflections (see Annexure 4). Fieldnotes capturing descriptive observations, verbatim speech, and researcher reflections were written immediately after each session, yielding over 100 pages of observational data. Team members maintained reflexive journals to document positionality and emotional responses.

We then moved to interviews, followed by FGDs. Semi‐structured topic guides for interviews and FGDs were developed by the research team based on the objectives of the study, the existing literature, and the experiences of the interdisciplinary research team (composed of anthropologists, sociologists, and psychiatrists). The topic guides for SSIs (Annexure 5) and FGDs (Annexure 6) covered topics such as the lived experiences of persons with FEP, explanatory models of the illness and treatment preferences, experiences of spiritual and traditional healings, access to healthcare, and views about delayed diagnosis and treatment, etc. The topic guides for SSIs were also piloted with two participants prior to data collection and refined for clarity and cultural appropriateness. All interviews and FGDs took place in the local language (Pashto) and were audio‐recorded with the informed consent of participants.

Prior to and during data collection, we conducted community engagement for about 6 months to build trust and understand local perspectives on mental illness and healing. This included implementing a Patient and Public Involvement and Engagement (PPIE) framework via a Lived Experience Advisory Panel (LEAP) of patients, carers, TSHs, and community representatives. LEAP helped in designing the topic guides and helped in the recruitment of participants for this study. Our community engagement also included informal visits to TSHs' consultation spaces to observe practice and build familiarity. Our community engagement activities helped in report‐building and enhanced the quality of interaction/observation at TSHs' consultation spaces. A detailed account of community engagement initiatives is reported separately in a forthcoming peer‐reviewed paper (accepted for publication by *Research Involvement and Engagement*).

### 2.6 Data Analysis

Data analysis was conducted manually without qualitative software. All interviews, FGDs, and fieldnotes were transcribed verbatim in Microsoft Word. We adopted a Framework Analysis approach (Goldsmith 2021), combining deductive and inductive coding. Analysis proceeded in three stages. In the first stage, two members of the research team independently read and inductively coded a subset of three observation fieldnotes transcripts to identify initial codes. This preliminary coding, resulting from inductive reading and informed by the study objectives, generated an initial thematic framework. In the second stage, the framework was applied systematically to all remaining transcripts. Fieldnotes and interview/FGD transcripts were indexed separately in two Excel spreadsheets to maintain source distinction and allow comparison. The framework remained flexible to accommodate emergent themes during coding of subsequent data. In the third stage, the field team members who collected that data independently entered the data into the framework, using relevant verbatim quotes, while a senior researcher (NS) conducted data cleaning and cross‐checked the entries to ensure consistency, accuracy, and analytical rigour.

Interpretation was guided by the ‘Pathways to Care’ framework (Rogler and Cortes 1993), which directed attention to sequences of help‐seeking, triggers for service selection, and structural enabling or constraining factors. We iteratively compared fieldnotes and interview/FGD datasets to examine convergence, divergence, and silence. Discrepant cases were retained as analytic opportunities rather than reconciled. Analytic decisions, including coding discrepancies and framework revisions, were resolved through team discussion. This multi‐source approach enabled triangulation of reported accounts with observed practices, strengthening the credibility, depth, and contextual validity of the findings.

### 2.7 Rigour and Trustworthiness

Methodological rigour and trustworthiness were ensured using Lincoln and Guba's criteria (Lincoln and Guba 1985). Data were collected by gender‐matched teams: male participants by two male researchers (SMUS, ITA) and female participants by two female researchers (SH, SFJ), all with at least master's degrees in social sciences and public health with qualitative research experience. All team members were insiders, native Pashto speakers with English fluency. Reflexive journals and team debriefings addressed researchers' positionality and power dynamics with TSHs and families. Pashto interviews were transcribed verbatim and translated into English by bilingual team members. Back‐translation of 10% of transcripts checked for meaning equivalence, with discrepancies resolved by consensus. Data were triangulated across participant groups and supplemented with fieldnotes. Prolonged and repeated community engagement with TSHs and peer debriefing with senior psychiatrists in the team (SF, MMA, MFK) supported interpretation and enhanced credibility. Member checking was not feasible due to stigma and literacy constraints.

### 2.8 Ethical Considerations

The study received ethical approval from Keele University Ethical Review Panel (ref: MH210177), Khyber Medical University Ethical Review Board (ref: DIR/KMU‐EB/IG/001005) and National Bioethics Committee Pakistan (ref: 4‐87/NBC‐840/22/621). All ethical considerations were adequately followed, including informed consent, ensuring the removal of participants' identifying features and confidentiality.

## 3 Results

### 3.1 Socio‐Demographic Characteristics of Study Participants

Characteristics of the TSHs selected for the ethnography (*n* = 15) are given in Annexure 1. Most TSHs identified and recruited in this study were male, indicating that spiritual healing is a masculine domain in the local culture. Due to cultural sensitivities, we were able to find only three female TSHs who were willing to participate in the study. All of the TSHs were well‐known in the community for their speciality of healing victims of ‘spirit possession’. TSHs were largely receptive to biomedical collaboration for psychosis care. Of 15 TSHs who participated in the focused ethnography, 13 endorsed referral partnerships. Resistance, where present, reflected concerns about state regulation and trust issues.

Characteristics of the young people with FEP (*n* = 12) and their carers (*n* = 14) selected for the interviews are given in Table 1 and Annexure 2, respectively. On average, young people with psychosis had visited three TSHs for treatment before coming to the hospital to seek psychiatric treatment. Most participants and caregivers described their households as having limited financial means, noting particular difficulty paying for hospital‐based treatment and costs related to travel and accommodation in Peshawar. Characteristics of the FGDs' participants are described in Annexure 3.

**TABLE 1:** Characteristics of young persons with psychosis selected for interviews (*n* = 12).

Characteristics		Number
Gender	Male	08
	Female	04
Age	14–16	02
	17–20	03
	21–25	07
Marital status	Married	02
	Unmarried	10
Location	Rural	08
	Urban	04
Duration since first diagnosed	Less than 6 months	04
	6 months to 1 year	04
	1–2 years	03
	Above 2 years	01
Visit to TSHs for treatment	Yes	11
	No	01
Number of TSHs visited	0	01
	1–2	06
	3–4	05

### 3.2 Socio‐Cultural and Health System Contexts Contributing to Longer DUP

Our analysis identified three mechanisms that structure pathways to care and prolong DUP: (1) Moral‐spiritual explanatory models that legitimise TSHs as first contact; (2) Stigma as reputational governance leading to concealment of illness; and (3) Structural exclusion that renders psychiatry less accessible.

#### 3.2.1 Religio‐Spiritual and Moral Frameworks as First‐Line Explanatory Models

We found that pathways to care were initially organised around moral and religio‐spiritual frameworks, not by biomedical explanation. Two dominant but interrelated models shaped interpretation: firstly, TSHs and some carers linked the onset of psychological distress to periods of life transition (such as puberty, marriage, sudden death of a relative) when the individuals are considered highly vulnerable and morally weak. During ethnographic fieldwork, one TSH explained the perceived causes and timing of spirit affliction:

You see, Allah has created a balance in life, a harmonious relationship with nature. However, sometimes the balance is disturbed, such as when people commit a grave sin. Here come ‘da ruhani tawazun masail’ (‘Issues of spiritual imbalance’). (Fieldnotes, Ethnography with TSH)

The TSH has established a cosmology of illness by explaining that health is divinely ordained and a moral problem, not a random biomedical phenomenon. As per his explanation, excess and transgression create vulnerability leading to possession/psychosis. Another TSHs explained that ‘spiritual treatment primarily aims to restore balance and harmony with nature, and between the body and soul’ (Fieldnotes, Ethnography with TSH). This TSH, and all other TSHs included in this study, always called mental health issues as *‘ruhani’* (spiritual) illness, not psychological, mental, or psychiatric. This emphasis on ‘spiritual’ dictates treatment: restore balance through ritual, not pills. We also noted that TSHs tried to claim authority in their field by emphasising that psychiatry does not and cannot understand the *‘ruhani realm’* as this form of knowledge lies outside the biomedical hierarchy. By naming the problem, TSHs legitimise themselves and delegitimise doctors as providers of first‐contact care.

Our ethnographic fieldnotes show that TSHs use a variety of different spiritual interpretations of psychosis:

There are four major sub‐types of spiritual explanations: Jadoo (magic), Jinnat (spirit possession), Nazar (evil eye), and Taveezoona (amulets). All of these have different manifestations and causes, yet all have the involvement of supernatural entities, afflicting the individual. Each of these types have their own sub‐types and their own spiritual treatment. (Fieldnotes, Ethnography with TSH)

Families frequently attributed onset of psychosis to the influence of *jinnat or taveezona* positioning the problem outside the individual but within the spiritual domain. This model mandated consultation with TSHs as first‐line healers.

For a long time, we believed it was because of ‘saya’ (spirits) or ‘taveezona’ (amulets) but later after two years or so we came to know that this is some mental illness. (Interview, female carer)

Here, the diagnosis of illness as *‘saya’* logically prescribes spiritual rituals (*taveez*, *dam (holly breath)*, and *shrine* visits), and inherently delay biomedical care. Psychiatric care becomes permissible only after ritual interventions are exhausted. The two‐year gap reflects the time needed to test and disconfirm the spiritual hypothesis, not necessarily ignorance of alternatives.

Upon the recommendations of various people, we visited spiritual healers and shrines of different villages …. Later, a relative advised us to show the patient to a doctor. Then we came here (i.e., hospital). (Interview‐female carer)

A related but different kind of moral interpretation also surfaced a number of times during interviews with carers. Aggression and disorganisation among adolescents were simultaneously read through a developmental‐moral lens. One carer, for example, narrated that:

When he started throwing things around and became aggressive, we thought he was just ‘hasay mast shaway de’ (‘spoiled by excessive youthful energy’) and acting out because of his growing age. (Interview, male carer)

Framing psychosis as ‘*mast’* (excessive youthful energy) normalise disruption as temporary and age‐appropriate behaviour, preserving family honour by avoiding the stigma of ‘madness’. This moral control strategy actively defers the problem and unless the behaviour sufficiently escalate beyond culturally acceptable explanation, it is not recognised as illness.

By contrast, we found that participants having previous experience of psychosis in their families were more likely to seek biomedical care:

We knew it was a mental illness because we had a similar experience before… so we brought him here to the hospital. (Interview, male carer)

This divergence shows that ‘understanding’ reflects access to a culturally legitimate psychiatric model. Prior experience reclassifies behaviours from ‘*jinnat*’ or ‘*saya*’ to ‘mental illness’, bypassing ritual sequencing.

As can be seen from the above excerpts, spiritual healing precedes biomedical care in most cases. All but one of the 12 persons with psychosis who participated in this study had visited spiritual healers before seeking hospital‐based care, and most of the carers spoke positively of the TSHs. Our ethnographic observations demonstrated that TSHs are usually humble, soft‐spoken, and caring when interacting with patients and carers (Fieldnotes, Ethnography with TSHs). For instance, they keenly listen to their problems and use affirmative religious language*: ‘Allah will give health’* and *‘If God willing, you will get well’*. Their low profile makes them easily approachable, where carers and patients feel comfortable.

On the other hand, while most of the carers and young persons with psychosis spoke positively of biomedical professionals, some of them described doctors as arrogant and distant, citing rushed consultations and poor communication.

The doctors walk with their hands in their pockets. They have attitude problems… ‘Dam Darood’ (spiritual healing) is cheap and effective, while a doctor is very expensive. … Doctors are very ‘takkabur’ (arrogant). (Interview, male carers).

Anthropological literature has established that in many traditional and ancient societies, illness is viewed as a disruption in the relationship between the individual, the spiritual realm, and the community (Greenway 1998). The framing of psychosis as a moral imbalance or spirit possession is what the anthropologist Kleinman terms ‘explanatory models’ (Kleinman 1978)—coherent systems that identified cause, dictated remedy, and deferred biomedical classification. Delay to psychiatry is built into the model. No clear referral pathways exist because TSHs and psychiatrists occupy different epistemic worlds. They are not ‘competing’; they treat different problems.

#### 3.2.2 Stigma as Social Regulation: Concealment, Ridicule, and the Management of Family Honour

Our data shows that stigma of mental health disorder operated not merely as a negative societal attitude, but as a system of moral‐social regulation that governed disclosure, help‐seeking, and household reputation. Several participants described that ‘people laugh’, ‘ridicule’ or ‘make jokes’ about the condition of the patients, and sometimes the patients (and family) are subjected to theological condemnation that positioned the sufferer as morally culpable:

We heard people say, ‘Due bande da Allah qahar nazil sho’ (God's wrath has befallen them). People make fun of them and make jokes. People tease the mentally ill to get themselves entertained. (FGD, male carer)

Here, stigma functions as labelling and the phrase *‘da Allah qahar’* (God's wrath) relabels distress from misfortune to divine punishment, morally slurring the individual and, by extension, the family.

Anticipating such public backlash, carers engaged in what sociologist Erving Goffman (Goffman 1963) calls ‘information control’: they actively try to conceal the issue to prevent a ‘spoiled identity’ from infecting the family:

We were worried about him, but we tried to hide his illness because then people would gossip about it and say, ‘Halak lewanay shave de’ (the person has gone mad). (Interview, male carer)

People usually hide such illnesses to avoid shame. To be honest, being a mother, I tried to hide and also directed my kids not to speak about the illness at home. (Interview, female carer)

Once applied, the Pashto label ‘*lewanay*’ (mad) functions as a ‘master status’ as it prevails all other social identities and justifies exclusion. Concealment therefore is used to protect not only the individual but collective honour of the family. The close association between madness and psychiatry works as barriers in the way of early intervention in psychosis. Avoidance of psychiatry services seems to be a logical strategy to manage stigma, not merely a result of ignorance.

Participants also described how mental health stigma ‘punishes the victim twice’; first, the direct burden of illness, including symptoms, disability, and distress; second, social consequences like blame, shame, exclusion, and delayed care. In Pakistan, families sometimes confine relatives with severe mental illness at home to avoid shame and embarrassment. Some are also subjected to physical abuse by family members or traditional healers.

The family confine the person who has mental illness because of stigma. Sometimes, they are locked up with chains. … Specifically, girls with mental health issues are not allowed to go out. (FGD, community member)

A gendered pattern was noted with respect to stigma as shame and loss of honour were associated particularly more with women having mental health issues. For young women, behavioural changes are often concealed due to concerns about marriage prospects and family honour.

There's a greater shame for girls because of mental illness. They are not considered suitable for marriage and face difficulty finding suitable marriage proposals. (FGD, community members)

TSHs narrated that women often resisted mental illness attributions, preferring spiritual explanations.

A young woman came to me and claimed she saw ‘jinnat’. I found no effects of jinn and advised her to see a psychiatrist. She got angry: ‘Wale za lewane em sa’ (Do you think I am mad?) (Fieldnotes, Ethnography with TSH).

Families actively managed disclosure for women with psychosis due to concerns about stigma and marriage prospects. As one TSH explained:

Interestingly, female patients and their relatives contact us before the visit. They request we keep the illness hidden so nobody knows. (Fieldnotes, Ethnography with TSH).

This pre‐emptive secrecy shaped pathways, with TSHs preferred over medical clinics to avoid social exposure. It was also revealed that some people with mental illness are abandoned by their family members due to stigma. Two of the participants of this study—both were married females suffering from psychosis—revealed that their in‐laws did not support them in getting treatment. Instead, they were supported by their parents and brothers. In one case, ‘the in‐laws declared the patient as ‘pagal’ (insane) and did not want to be with her in the hospital’ (Interview, female carer).

Drawing on Goffman's concept of ‘spoiled identity’ (Goffman 1963), participants described how psychosis threatened both individual and family standing, triggering strategies of concealment and avoidance of psychiatric services. Under these conditions, TSHs become structurally preferable as the ritual healing usually occurs in private, religious spaces that carry no psychiatric label, allowing families to seek help while maintaining public face.

#### 3.2.3 Structural Exclusion: Poverty, Distance, and Clinical Neglect of Psychosis in Primary Care

While religio‐spiritual frameworks and stigma positioned TSHs as culturally safe first contact, the formal health system's structural neglect rendered biomedical care less accessible, consolidating therapeutic dependence on TSHs.

Most FEP individuals and carers who participated in this study came from remote areas of Peshawar and surrounding districts. For such families, psychiatry was absent by design: no services, no specialists, and no affordability. This structural exclusion was explained by a young person currently on treatment:

We belong to a faraway village where there are no such treatment facilities … no mental illness doctor in the area. It was difficult for us to come from the village to Peshawar. (Interview, young person with psychosis)

In the absence of psychiatry at the primary level, families were structurally positioned to use proximate TSHs: ‘Young people… may consult a TSH because of their proximity to a mosque, madrassah, shrine, or other religious places’ (Fieldnotes). TSHs thus fill a vacuum created by state disinvestment, not by cultural preference alone.

All but one of the carers described themselves as belonging to poor households and pointed out their inability to afford the high treatment costs at the tertiary care hospital:

For poor people like us, it is very difficult to get treatment. It costs us very much to deal with his treatment, medicines, and meet other expenses, say, travel, food and the like… (Interview, male carer)

First, you cannot find a ‘demaghi doctor’. If you can find one, it is very expensive. (Interview, female carer)

Here, cost systematically disadvantages the poor as treatment requires not only consultation fees but cumulative expenses of transport, food, and lost wages. Under these conditions, TSHs become economically rational first‐line care because they are ‘culturally more acceptable, easily accessible, and financially affordable’ (Fieldnotes).

We found that primary care physicians are the second most common point of contact for psychiatric patients in Pakistan:

Initially, we visited ‘dam‐darood wala’ (TSHs), and then we visited a local doctor (PCP) who gave medicines, but no effect was observed, and the actual illness could not be known/diagnosed for a long time. (Interview, female carer)

Even when families reach local health facilities, mental health is deprioritised within primary care itself. We found that primary care physicians (PCPs) lacked time, training, and institutional mandate to manage psychosis, reproducing exclusion at the point of service.

The truth is that most primary care physicians are not trained to deal with mental health issues. The PCPs cannot identify or diagnose mental illnesses. I think in our setup (i.e. clinic), 80% of the mental health issues go undiagnosed. (FGD, Primary Care Physician)

Thus, the absence of skill is not an individual deficit but a system signal: mental health remains ‘low priority’ in resource allocation, training, and accountability. Collectively, geographic distance, poverty, and incapacity of primary care produce therapeutic abandonment: a system organised to make psychosis untreatable for rural, poor families. As a result, pathways sequence toward TSHs.

## 4 Discussion

Viewed through the ‘Pathways to Care’ framework, help‐seeking was shaped by a religio‐spiritual explanatory model that made TSHs the culturally legitimate first contact, while stigma‐driven concealment of illness and structural barriers like distance and absent referral pathways delayed biomedical care, prolonging the duration of untreated psychosis. Progression to psychiatric services thus depended on navigating cultural and systemic factors, not symptom severity alone. The results of the study resonate with the findings of a recent study on barriers to the treatment of mental health disorders in LMICs, according to which the three most frequently reported barriers include a preference for traditional and alternative treatments (33.33%), stigma (25%), and low level of mental health literacy among the people (25%) (Saade et al. 2023).

Reliance on TSHs in FEP management reflects Pakistani society's religious and socio‐economic context as well as structural gaps in formal healthcare accessibility. Given the sufficient evidence provided by this and other studies on the topic (Arooj 2023; Anjorin and Hassan Wada 2022), we can argue that the poorer the people and the healthcare system, the greater the reliance on TSHs as the first‐contact providers. TSHs articulated psychosis through a cosmology of divine balance, where symptoms signalled issues of spiritual disequilibrium—*‘ruhani tawazun’*—triggered by moral transgression or dislocation. Within this framework, distress is interpreted as metaphysical, positioning ritual purification as the appropriate first‐line care. Psychiatric intervention becomes legible only after spiritual remedies are exhausted, sequencing delays as a rational outcome of pluralistic healing logic.

The care provided by TSHs is complementary to societal belief systems and ‘*ruhani* treatment’ is often more acceptable to the patients and carers within that context and less stigmatised as a whole. Indeed, some patients are willing to be declared ‘possessed’ but not willing to be labelled ‘psychotic’. In ‘possession’, the patient is usually viewed as a ‘victim’ of supernatural spirits which are beyond the control of ordinary human beings. Thus, the ‘possessed’ person deserves the sympathies of people, not ostracism and humiliation. A recent study found that in Pakistan, in some cases, ‘possession is considered power’ (Ahmad et al. 2023) as the possessed individual is believed to be able to perform extraordinary tasks (such as speaking a foreign language, locating stolen items, lifting heavy objects, etc., with the help of *Jinnat*). This is not the case with patients declared as psychotic.

Findings illustrate how stigma operated along gendered lines to shape both the interpretation of symptoms and pathways to care. TSHs reported pre‐emptive requests from relatives of female patients to ‘keep the illness hidden so nobody knows’, reflecting awareness that public knowledge of psychosis could render women unmarriageable. This aligns with work in South Asia showing that mental illness stigma for women is mediated through kinship and marital economies, where a daughter's illness threatens not only her marriage prospects but the family's social standing (Sahai‐Siddiqui 2025). Assumptions that TSHs simply ‘delay’ care also overlook their role in providing culturally legitimate, confidential care for women who cannot afford public exposure. It is worth noting that the gendered patterns reported here emerged inductively and warrant dedicated research examining how gender intersects with marital status, age, and socioeconomic position to shape pathways.

Although PCPs can play a significant role in reducing DUP, they do not possess the necessary training to diagnose common mental disorders nor do they have the time required for such investigation and referral. This is consistent with the findings of a study in Peshawar which shows that ‘the knowledge and practice skills of general physicians were below average by medical standards regarding diagnosis and treatment of psychosis’ (Irfan et al. 2015). Psychiatric services, on the other hand, are not easily accessible and available in Pakistan, like most other LMICs.

Besides the barriers, we also found some facilitators for early diagnosis and treatment of psychosis. These include referral to services by a family member, schoolteacher, or PCP. Interestingly, some patients were also advised by TSHs to see a doctor, which suggests the possibility of utilising the services of TSHs within primary healthcare to ensure early diagnosis and prompt treatment of FEP. The majority of TSHs selected for ethnography (13 of 15) expressed willingness to work in collaboration with PCPs and psychiatrists. However, structural barriers within formal healthcare constrain TSH–PCP collaboration beyond interpersonal willingness. PCPs cited a lack of time and training, while TSHs described having no knowledge of pathways to refer. Discussion with PCPs also revealed that non‐recognition and the issue of legitimacy create legal risk that discourages engagement, reinforcing biomedical hierarchy in which TSHs are excluded from clinical spaces and regarded as non‐scientific by biomedical providers. While a detailed discussion on TSHs‐bio‐medical collaboration is beyond the scope of this paper, a systematic review synthesising existing models of such collaboration, along with their effectiveness in early identification and treatment of first‐episode psychosis, would be a valuable next step.

These findings offer insights for Pakistan where traditional and spiritual healers operate alongside under‐resourced mental health services. Transferability to other LMIC contexts may be considered where similar pluralistic health systems, strong family decision‐making structures, and limited psychiatric services exist.

### 4.1 Strengths and Limitations of the Study

This study has several strengths. First, sustained community engagement enabled access to a population often excluded from mental health research, generating first‐hand accounts from TSHs alongside patient and caregiver perspectives. Second, triangulation across stakeholders strengthens credibility and illuminates divergent understandings of help‐seeking. Third, the study provides rich contextual data from Pakistan, an understudied setting where pluralistic healing is normative, addressing gaps in the predominantly HIC‐focused DUP literature. Finally, the ethnographic approach captured process and meaning‐making rather than static variables, offering nuanced insights into how delays are experienced and navigated.

On the other hand, several limitations of this study should be noted. First, young persons with psychosis and carers were recruited from a single tertiary hospital in Peshawar, which may limit transferability of findings to other regions of Pakistan. Second, our sample excludes individuals with psychosis who never access biomedical care, and thus cannot capture pathways of those entirely outside the health system. Third, although the translation for interviews from Pashto to English was conducted by bilingual researchers, some nuances of meaning may have been lost. Fourth, as a qualitative study, we did not measure DUP quantitatively. Findings reflect participants' perceptions of delays to biomedical care and potential influences on help‐seeking pathways, and should not be interpreted as evidence of measured DUP reduction. Lastly, as with all qualitative research, findings represent the perspectives of participants at a specific time and place and may not be generalisable to other areas inside or outside Pakistan.

## 5 Conclusion

Families in Pakistan navigate multiple forms of care after the onset of psychosis, with traditional and spiritual healers often serving as first‐contact providers. Religio‐moral and spiritual explanatory models, stigma, and fragmented access to mental health services shaped help‐seeking pathways and contributed to perceived delays in accessing biomedical care. These accounts suggest that efforts to support timely care may benefit from engaging with the realities of pluralistic help‐seeking. TSHs are easily available, accessible, affordable, and culturally more acceptable as compared to psychiatric services. This points to the potential value of exploring culturally acceptable and context‐appropriate strategies for increasing access to mental healthcare in low‐resource settings, which includes collaboration with TSHs. Future research could examine whether and how collaborative models are acceptable to TSHs, families, and biomedical care providers in this setting, and what ethical and practical safeguards would be required. While context‐specific, findings of this study may offer theoretical insights for other low‐resource settings where TSHs play a central role in mental healthcare and where similar socio‐cultural and health system factors exist.

## Funding

This study is jointly funded by the UK Medical Research Council (MRC) and the Foreign Commonwealth and Development Office (FCDO) under the MRC/FCDO Concordat agreement, together with the Department of Health and Social Care (DHSC) (Grant number: MR/T040378/1).

## Conflicts of Interest

The authors declare no conflicts of interest.

## Supporting information


**Annexure 1.** Characteristics of traditional and spiritual healers (*n* = 15) selected for ethnography.
**Annexure 2.** Characteristics of carers selected for interviews (*n* = 14).
**Annexure 3.** Characteristics of participants selected for focus group discussions (FGDs).
**Annexure 4.** Observation framework for ethnography with traditional and spiritual healers (TSHs).
**Annexure 5.** Topic guides for semi structured interviews (SSIs).
**Annexure 6.** Topic guides for focus group discussions (FGDs).

## Data Availability

The data supporting this study's findings are available on request from the corresponding author, S.F. The data are not publicly available due to privacy and ethical restrictions.
