# Supplementary material for: Untreated Psychosis and Healthcare‐Seeking Behaviour in Pakistan: A Qualitative Study With Young People, Carers, Traditional Healers, and Healthcare Professionals
Source: Early Interv Psychiatry. 2026 Aug 4;20(8):e70233. doi: 10.1111/eip.70233 (PMC13435109; doi:10.1111/eip.70233)
Supplement: Supplementary file 1 [file EIP-20-0-s001.docx]

**Table of contents**

[**Annexure 1: Characteristics of traditional and spiritual healers (n=15) selected for ethnography**	2](#_Toc232065458)

[**Annexure 2: Characteristics of carers selected for interviews (n=14)**	3](#_Toc232065459)

[**Annexure 3: Characteristics of participants selected for focus group discussions (FGDs)**	4](#_Toc232065460)

[**Annexure 4: Observation framework for ethnography with traditional and spiritual healers (TSHs)**	5](#_Toc232065461)

[**Annexure 5: Topic guides for semi structured interviews (SSIs)**	9](#_Toc232065462)

[**Annexure 6: Topic guides for focus group discussions (FGDs)**	15](#_Toc232065463)

# **Annexure 1: Characteristics of traditional and spiritual healers (n=15) selected for ethnography**

| **Variable** | | **Number** |
| --- | --- | --- |
| **Gender** | Male | 12 |
|  | Female | 03 |
| **Age** | 30 and below | 02 |
|  | 31-50 | 06 |
|  | 51-70 | 05 |
|  | Above 70 | 02 |
| **Education** (years of schooling) | 14 years and above | 05 |
|  | 10-12 years | 03 |
|  | Below 8 years | 05 |
|  | No formal schooling | 02 |
| **Location** | Rural | 09 |
|  | Urban | 06 |
| **Specialisation / Famous for** | Only spirit possession | 03 |
|  | Spirit possession & other spiritual illnesses | 12 |
| **Fee /charges for healing** | No fee at all | 03 |
|  | No specific amount (clients’ choice) | 09 |
|  | Rs 50- 2000 | 03 |
| **No. of patients seen per week** | 15-30 | 07 |
|  | 31-45 | 02 |
|  | 45-60 | 01 |
|  | Above 60 | 01 |
| **Years of training received to learn spiritual healing** | No formal training | 02 |
|  | 1-4 years | 03 |
|  | 5-10 years | 06 |
|  | 20 and above years | 04 |
| **Willingness to collaborate with primary care and psychiatrists** | Yes | 13 |
|  | Reluctant | 02 |

# **Annexure 2: Characteristics of carers selected for interviews (n=14)**

| **Variable** | | **Number** |
| --- | --- | --- |
| **Gender** | Male | 07 |
|  | Female | 07 |
| **Age** | 30-50 | 07 |
|  | 51-70 | 07 |
| **Education**  (years of schooling) | 14 years | 03 |
|  | 12 years | 0 |
|  | 10 years | 04 |
|  | No formal schooling | 07 |
| **Occupation** | Unemployed | 02 |
|  | Unskilled labour | 04 |
|  | Government servant | 02 |
|  | House wife | 06 |
| **Location** | Rural | 11 |
|  | Urban | 03 |
| **Relationship with the patient** | Father | 04 |
|  | Mother | 06 |
|  | Brother | 02 |
|  | Other relatives | 02 |

# **Annexure 3: Characteristics of participants selected for focus group discussions (FGDs)**

| **Category** | **Gender** | **Education** | **Occupation** | |
| --- | --- | --- | --- | --- |
| FGD with Psychiatrists | Male | FCPS | Psychiatrist | |
|  | Male | FCPS | Psychiatrist | |
|  | Male | FCPS | Psychiatrist | |
|  | Male | FCPS | Psychiatrist | |
|  | Male | MCPS | Psychiatrist | |
| FGD with Primary Care Physicians | Male | MBBS | Primary care physician | |
|  | Male | MBBS | Primary care physician | |
|  | Male | MBBS | Primary care physician | |
|  | Male | MBBS | Primary care physician | |
|  | Female | MBBS | Primary care physician | |
|  | Female | MBBS | Primary care physician | |
|  | Female | MBBS | Primary care physician | |
| FGD with Community Members | Male | MBA | Traditional & spiritual healer | |
|  | Male | Bachelor | Government servant | |
|  | Male | Master | Self-employed/small business | |
|  | Male | Bachelor | Employee in the private sector | |
|  | Female | Master | Government servant | |
|  | Female | Master | Healthcare worker | |
|  | Female | Bachelor | Housewife | |
| FGD with Traditional and Spiritual Healers | Male | Bachelor | Seminary teacher & spiritual healer | |
|  | Male | Middle | Seminary teacher & spiritual healer | |
|  | Male | Middle | Traditional & spiritual healer | |
|  | Male | Matric | Seminary teacher & spiritual healer | |
|  | Male | Bachelor | Private business & spiritual healer | |
|  | Male | Middle | Retired government official & healer | |
|  | Male | Bachelor | Private business & spiritual healer | |
| **Category** | **Gender** | **Education** | **Occupation** | **Relationship with patients** |
| FGD with  Male Carers | Male | Matric | Daily wage labour | Brother-in-law |
|  | Male | Matric | Self-Employed | Father |
|  | Male | Intermediate | Daily wage labour | Brother |
|  | Male | Master | Government servant | Father |
|  | Male | Bachelor | Daily wage labour | Uncle |
|  | Male | Intermediate | Government servant | Father |
| FGD with Female Carers | Female | Primary | Housewife | Mother |
|  | Female | Primary | Housewife | Mother |
|  | Female | Uneducated | Housewife | Mother |
|  | Female | Uneducated | Housewife | Mother |

# **Annexure 4: Observation framework for ethnography with traditional and spiritual healers (TSHs)**

1. Name/Identification the ethnographic setting: _______________________________

2. Name of ethnographer(s): _______________________________________________

3. Date: ____________________ Time: Start: ______________ End: ______________

**Notes for Ethnographer/s**

- This tool includes a list of topics/issues for you to consider in the observation. Please write short descriptions of what you observe. Later on, these short notes should be elaborated as soon as possible.
- During the observation, detailed attention should be paid to the ‘who, what, where, and when’ of the different processes taking place in the ethnographic setting.
- After these more general observations, the focus of the observation should shift to the interactions between people in the social setting (TSH, patients, interaction among patients and carers etc.).
- This is not JUST a questionnaire. Rather, it is a set of topics to guide the observation process.
- The topics listed below are not final. You can record any other important event, process, or fact that you might come across during the observation. This topic guide will be updated throughout fieldwork due to the iterative nature of ethnographic research.
- Whenever possible, try to engage with the TSH and other people in the setting for clarification and more details on a specific aspect of an observed phenomenon.
- Where appropriate, make sure that you have necessary measures in place to protect yourself from any physical/medical hazards during the ethnography.

1. **The Social Setting**

| **S. No** | **Themes/ Activities** | **Notes** |
| --- | --- | --- |
| **1** | Location (Home / Hujra / Mosque) |  |
| **2** | The nature of the Social Setting (indoor/outdoor, comfortable, spacious, accessible or secret, etc.) |  |
| **3** | Physical elements (building, furniture, spiritual artifacts, etc.) |  |
| **4** | People in the setting (the presence of other people, privacy etc.) |  |
| **5** | The waiting area (space, facilities, number of people and social interaction) |  |
| **6** | Written, visual instructions for patients and general people in the setting |  |
| **7** | Major activities going on in the setting |  |

2. **The Traditional and Spiritual Healer**

| **S. No.** | **Themes/Activities** | **Notes** |
| --- | --- | --- |
| **1** | Age and Gender |  |
| **2** | Primary identity and specialization (traditional herbalist, spiritual, religious, magician) |  |
| **3** | Nature of training received (duration, formal, informal) |  |
| **4** | Appearance (clothing etc.) |  |
| **5** | Methods used for dealing with illness/diagnosis of illness (What terns TSH use for explaining symptoms to patients or carers |  |
| **6** | Process and procedure of healing |  |
| **7** | Tools and techniques used for healing |  |
| **8** | The attitude of TSH towards the patient (indifferent, sympathetic, friendly) |  |
| **9** | Common words used during the healing process |  |
| **10** | Post-healing counselling/ advice |  |
| **11** | Fee (*shukrana -*gratitude money) demanded / received /negotiated |  |

3. **The Patient(s)**

| **S. No.** | **Activities** | **Notes** |
| --- | --- | --- |
| **1** | Age, gender, physical appearance, apparent socio-economic status |  |
| **2** | First visit/follow-up visit |  |
| **3** | Apparent physical and psychological health of the patient |  |
| **4** | Patent’s description of their illness (words and phrases used) |  |
| **5** | Willingness / consent of patients in getting the treatment |  |
| **6** | Local words being used to refer to illness, physical and psychological condition, healing, etc. |  |
| **7** | Idioms and expressions used by patients to describe their social, physical and psychological wellbeing |  |
| **8** | Patients’ experiences of receiving various types of treatments received so far (traditional/spiritual/medical) |  |
| **9** | Barriers to seeking treatment – economic, social, and cultural (stigma, poverty etc) |  |
| **10** | Feelings or experience suggestive of stigma among the patients/attendants |  |
| **11** | Signs and symptoms of mental illness among the patients |  |
| **12** | Expressions of patient before, during and after the healing process |  |

4. **Nature of Interaction between the Patient, Carers and TSH**

| **S. No.** | **Themes / Activities** | **Notes** |
| --- | --- | --- |
| **1** | Nature of interaction between TSH and the patients/carers (formal, informal, personal, impersonal, general) |  |
| **2** | Duration of the interaction (Give approximate time like 5-minute, 10 minute, ½ hour) |  |
| **3** | The major focus of the TSH during the interaction (investigation, verbal therapy, meditation, etc.) |  |
| **4** | Advice given to the patient / carers? |  |
| **5** | Whether a return appointment was booked |  |
| **6** | Nature of interaction between the TSH and the patient (cooperative, non-cooperative, coercive) |  |
| **7** | Any evidence of coercion, abuse, (verbal or physical towards patients from family member or carer or any other person. |  |

5. **The Parents/ Carers/Attendants**

| **S. No.** | **Themes / Activities** | **Notes** |
| --- | --- | --- |
| **1** | Age, gender, relations and number of carers |  |
| **2** | Apparent /self- reported socio-economic condition of the carers |  |
| **3** | The extent of involvement and role of patient’s relatives in the interaction |  |
| **4** | Carer’s narration of the history the disease and treatment obtained before |  |
| **5** | Carer’s motivation behind seeking the current treatment |  |
| **6** | Words and phrases used by the carers to explain the condition of the patient |  |
| **7** | Interaction among carers of various patients present in the social setting |  |
| **8** | Expressions of carers before, during and after the healing process |  |

6. **Any Other Relevant Observation**

| **S. No.** | **Themes / Activities** | **Notes** |
| --- | --- | --- |
| **1** | Most frequent topics discussed, vocabularies used, and questions raised in the social setting |  |
| **2** | Nonverbal behaviours of people – patients, attendants, other |  |
| **3** | Effects of ethnographers on the social processes in the setting |  |
| **4** | Any effects of ethnographers on the nature of interaction/therapeutic processes |  |
| **5** | TSH’s attitude/perception of biomedical treatment |  |
| **6** | Will TSH be willing to collaborate with formal healthcare (BHUs/mental health professionals) in referring patients with mental illness? What are their fears/apprehensions in this regards |  |
| **7** | Any other Observation |  |

# **Annexure 5: Topic guides for semi structured interviews (SSIs)**

**1. Topic guide for interview with parents and carers of young persons with psychosis**

**Demographic Information**

1. Name:	________________________________
2. Age:	________________________________
3. Gender:	________________________________
4. Location /Address:	________________________________
5. Occupation:	________________________________
6. Education:	________________________________
7. Family type:	________________________________
8. Marital Status:	________________________________
9. Socioeconomic status:	___________________________________

**Parent’s / Carer’s Perception and Description of Psychosis**

10. Would you please tell us something about the illness and mental health of your child / the patient?

- conditions and circumstances when he/she first experience psychosis?

11. What were the initial symptoms of his/her illness?

- What changes have you noticed in symptom of the patient over time?

12. For how long was patient suffering from the illness and how was he/she diagnosed?
13. What were the initial reactions of people and your family members when the patient experienced symptoms for the first time?

- How you and other family members initially interpreted the illness?
- How your perception of the illness changed over time?

**The Lived Experiences of Parents / Carers of Persons with Psychosis**

14. As a person who is taking care of the patient, how do you live your routine life?

- Effects on life of carers due to illness of the patient?
- Effects of education, work, social interaction with people?
- What changes have you noticed in your life after the illness of the patient?
- Who else in the family is usually taking care of the patient?

15. Did you notice any change in the attitude of people after child was diagnosed with psychosis?

- How your colleagues, friends and general people reacted towards you and your family after the illness of your child/relative?
- Any instance of stigmatized labels and expressions by people about the patients?

16. How often do you experience unusual behavior from the patient?

- Kinds of behaviors exhibited by the patients
- How do you usually cope with deal with the patient?

17. What aspect of the care of the patient is the most disturbing and tiring for you and how?
18. What do you suggest for a better care of a psychosis patient?

**Treatment History of the Persons with Psychosis**

19. Please share the various kinds of treatments the patient has gone through so far.

- Traditional and spiritual healing
- Self-Medication
- Folk / Herbal?
- Allopathic
- Psychiatry

20. If you have visited Traditional and /or Spiritual Healers for the treatment of the patient, who recommended them and what was your experience with such treatment?

- Any mistreatment by TSH?
- Consent of the patient in seeing TSH?
- Your satisfaction with the treatment of TSH?

21. Why do you think people visit TSH for the treatment of psychosis patients?
22. If the patient has received allopathic treatment, tell us about your satisfaction with such treatment.
23. If the patient has received psychiatric treatment, who referred him/her to the psychiatrist and how was your experience of such treatment?
24. In your experience, is it easy or difficult to take psychiatric treatment? (In terms of accessibility, financial cost, social acceptability, adherence etc.)
25. Who in your family is primarily deciding on the treatment of the patient?

- To what extent is the patient involved in such decision-making? if not, why?
- How can we increase the involvement of patients in decision-making regarding their treatment?

26. Based on your experiences of taking care of patient with psychosis, what would be your recommendations for the treatment of a newly diagnosed psychosis patient?

**Ways to Improve Early Detection and Treatment of Psychosis**

27. Elaborate upon how the illness of the patient was first detected and when?
28. How long it took between symptoms of the illness and proper psychiatric treatment of the patient?
29. In case treatment was delayed by at least a year, what were the main factors for the late consulting of psychiatrist?
30. In your opinion, how delayed treatment has affected the health of the patient?
31. If you are currently seeking psychiatric treatment for your patient, who referred you to this hospital/psychiatrist?

- How satisfied do you feel with the treatment process?
- How can we further improve the treatment process?

32. In your opinion, what social and cultural factors work as barriers to early treatment of psychosis in our society?
33. What do you suggest for the early detection and early treatment of psychosis?

**General Discussion and Closing Remarks**

34. Do you want to share with us anything about the illness/patients, the TSH, or anything related to the topic that you have not shared so far?
35. Do you have any questions from the researcher regarding the illness or its treatment?
36. Do you have any questions from the researcher regarding this research/interview?

Thank you very much for your time.

**2. Topic guide for interview with young persons with psychosis in Peshawar, Pakistan**

**Demographic Information**

37. Name:	________________________________
38. Age:	________________________________
39. Gender:	________________________________
40. Location /Address:	________________________________
41. Occupation:	________________________________
42. Education:	________________________________
43. Family type:	________________________________
44. Marital Status:	________________________________

**Patient’s Perception and Description of Psychosis**

45. Would you please tell us something about your illness and mental health?

- feelings and conditions when you first experience psychosis.
- initial symptoms of the illness?
- change in symptoms over time?
- since how long have you been suffering from the illness and how was it diagnosed?
- initial reactions of people and your family members when you suffered from the illness?
- how people around you initially interpreted your illness?

**The Lived Experiences of FEP Patients**

46. How do you live your routine life with the illness?

- How your life has been affected by the illness?
- Effects of education, work, and social interaction with people?
- What changes have you noticed in your life after the illness?

47. Did you notice any change in the attitude and behavior of people around you after your diagnosis?

- How do your colleagues and friends react towards you after your illness?
- Use of any stigmatized labels by people?

**History and Pathways Care of the Patients**

48. Please share your experiences of different kinds of treatments you have received so far.

- Traditional and spiritual healing
- Self-Medication
- Folk / Herbal?
- Allopathic
- Psychiatry

49. If you have visited Traditional and /or spiritual healers, who recommended them and what was your experience with such treatment?

- Any mistreatment by TSH?
- Consent of the patient in seeing TSH?
- Your satisfaction with the treatment of TSH?

50. If you have received allopathic treatment, tell us about your satisfaction with such treatment.
51. If you have received psychiatric treatment, who referred you to the psychiatrist and how was your experience of such treatment?
52. Is it easy or difficult to take psychiatric treatment? (In terms of accessibility, financial cost, social acceptability, adherence etc.)
53. How much say do you have in deciding the various types of treatments you have received so far?

- Who, in your opinion, should decide the various types of treatment for the patient – the patient or the parents/carers? Why?
- How can we increase the involvement of patients in decision-making regarding their treatment?

54. Based on your experience of going through the various types of treatments, what would be your recommendation for the treatment of a newly diagnosed psychosis patient?

**Ways to Improve Early Detection and Treatment of Psychosis**

55. Elaborate upon how your illness was first detected and when?
56. Who were the people who identified your illness as psychosis?
57. How long it took between detection of your illness and seeking help of a psychiatrist?
58. In case your treatment was delayed, what were the main factors?
59. In your opinion, how delayed treatment has affected your health?
60. If you are currently taking Psychiatric treatment, who referred you to this hospital/psychiatrist?

- How satisfied do you feel with the treatment process?
- How can we further improve the treatment process?

61. In your opinion, what social and cultural factors work as barriers to early treatment of psychosis in our society?
62. What do you suggest for the early detection and early treatment of psychosis among young people?

- Awareness among parents/young people
- Training of Healthcare providers

**General Discussion and Closing Remarks**

63. Do you want to share with us anything that you have not shared so far?
64. Do you have any questions from the researcher regarding your illness?
65. Do you have any questions from the researcher regarding this research/interview?

Thank you very much for your time.

# **Annexure 6: Topic guides for focus group discussions (FGDs)**

**1. Topic guide for FGD with traditional and spiritual healers**

Day and Date: __________________	Time: Start ________ End __________

Moderator: _____________________	Note-Taker: _____________________

Venue: ________________________	Total Number of Participants: _______

**Demographic Information (separate record of all participants to be obtained)**

- Name:	________________________________
- Age:	________________________________
- Gender:	________________________________
- Location /Address:	________________________________
- Occupation:	________________________________
- Education:	________________________________

**Perception about Spiritual Illnesses / Psychosis among Adolescents**

1. What are the most common types of mental illnesses experienced by adolescent patients between 14 and 25 years of age?
2. What are the demographic characteristics of people suffering from mental/spirit illnesses?

(age, gender, economic status, education, rural-urban, etc.)

3. What are the most common symptoms and signs of spirit possessions (or Jinnat)?
4. Why do you think adolescents are possessed by Jinnat?

**Techniques and Methods used by TSH for Treatment of Spiritual/mental Illnesses**

5. Would you kindly share some of the common techniques and methods you use for treatment of such patients?
6. How effective these treatment process and methods are?
7. Are there any side-effects of the treatment you provide to patients?

**Attitude of TSH towards Psychosis and Psychiatry**

8. How do you view the mental illness and modern psychiatry?
9. How often do you come across patients who you think should consult a psychiatrist? Do you refer such cases to a psychiatrist?

**Willingness to work with PCPs and Psychiatrists**

10. If given a chance, would you be willing to work in collaboration with doctors and psychiatrists?
11. Would you be willing to get some training from doctors and psychiatrists on mental illness? On what conditions, if any?
12. Before we close this discussion, is there another thing you want to share?

Thank you very much for your cooperation.

**2.** **Topic guide for FGD with primary care physicians in Peshawar, Pakistan**

Date: __________________	Time: Start ________ End __________

Moderator: _____________________	Note-Taker: _____________________

Venue: ________________________	Total Number of Participants: _______

**Demographic Information (separate record of all participants to be obtained)**

- Name:	________________________________
- Age:	________________________________
- Gender:	________________________________
- Specialization	________________________________
- Location /Address:	________________________________

**Perception about Psychosis among Adolescents**

13. Would you please share your perception about mental illness, especially psychosis among adolescents?
14. How to do you see the phenomenon of ‘spirit possession’ or ‘*Jinnat*’? How frequently do you come across such person?
15. Why do you think patients of psychosis usually seek treatment from traditional and Spiritual Healers (TSHs)?

**Stigma and Suggestions for Minimizing It**

16. To what extent is mental illness a stigma in our society and how does it affect the patient and their family?
17. The Duration of Untreated Psychosis (DUP) is much longer in poor countries like Pakistan as compared to developed countries. In your opinion, what could be the reason for the late detection and treatment of psychosis in our country?
18. What do you suggest to minimize stigma around psychosis and how to ensure early detection and treatment of the disease?

**Training needs of PCP Regrading Psychosis**

19. Being a doctor, how would you rate your own knowledge about psychosis and how do you usually deal with a psychosis patient?
20. Would you be willing to receive training in psychosis and help in the early detection and treatment of psychosis patients?

**PCP working in collaboration with TSH**

21. Would you be willing to work in collaboration with TSHs for early detection and treatment of psychosis among adolescents?
22. What, in your opinion, could be the best possible mechanism for establishing collaboration among TSHs and PCP?
23. Before we close this discussion, is there another thing you want to share?

Thank you very much for your cooperation.

**3.** **Topic guide for FGD with psychiatrists in Peshawar, Pakistan**

Date: __________________	Time: Start ________ End __________

Moderator: _____________________	Note-Taker: _____________________

Venue: ________________________	Total Number of Participants: _______

**Demographic Information (separate record of all participants to be obtained)**

- Name:	________________________________
- Age:	________________________________
- Gender:	________________________________
- Location /Address:	________________________________

**Perception about ‘Spirit Possession’ and TSHs**

24. Would you please share your personal and professional experience of dealing with patients suffering from mental health disorders?
25. What do you think about Traditional and Spiritual Healers and their methods of treating patients with mental disorders?
26. Why do you think patients of psychosis usually seek treatment from traditional and Spiritual Healers (TSHs)?

**Stigma and Suggestions for Minimizing It**

27. To what extent is mental illness a stigma in our society and how does it affect the patient and their family?
28. The Duration of Untreated Psychosis (DUP) is much longer in poor countries like Pakistan as compared to developed countries. In your opinion, what could be the possible reasons for the late detection and treatment of psychosis in our country?
29. What do you suggest to minimize stigma around psychosis and how to ensure early detection and treatment of the disease?

**Psychiatrists working with PCP and TSH**

30. Would you be willing to work in collaboration with PCPs and TSHs for early detection and treatment of psychosis among adolescents?
31. What, in your opinion, could be the best possible mechanism for establishing collaboration among TSHs and PCPs?
32. What kind of training do you think psychiatrists should provide to PCPs and TSHs for early detection and referral of psychosis patients?
33. Before we close this discussion, is there another thing you want to share?

Thank you very much for your cooperation.

**4.** **Topic Guide for FGD with parents/carers of persons with psychosis**

Day and Date: __________________	Time: Start ________ End __________

Moderator: _____________________	Note-Taker: _____________________

Venue: ________________________	Total Number of Participants: _______

**Demographic Information (separate record of all participants to be obtained)**

- Name:	________________________________
- Age:	________________________________
- Gender:	________________________________
- Location /Address:	________________________________
- Occupation:	________________________________
- Education:	________________________________

**Lived Experiences of Carers of Psychosis Patients**

34. How do you live your routine life with, and take care of, a psychosis patient?
35. Being a carer of a psychosis patient, what are some of the problems you have to face in day-to-day life?

**Symptoms and Behavior of Psychosis Patient**

36. Would you please describe some of the common symptoms expressed by your patients?
37. What are some of the ‘triggers or situations in which the patient starts behaving abnormally?

**Stigma and People's Perception of Psychosis**

38. Would you kindly share how the patient was first diagnosed and what was the reaction of family members towards the patient?
39. What is the general reaction of people in society and neighborhood regarding psychosis patient and the family?

**Late Detection and Treatment of the Patient**

40. Would you please share the treatment history of the patient and how he/she was ultimately brought to the hospital?
41. If you have consulted TSHs for the treatment of the patient, how would you describe your experience?
42. In your opinion, what are the causes responsible for delay in diagnosis and treatment of psychosis patients?
43. Based on your personal observations and experiences of being a carer of a psychosis patient, what do you suggest for early detection and treatment of psychosis among adolescents?

**TSHs Working with Primary Care Physicians**

44. Do you think it is possible and desirable for Traditional and Spiritual Healers to work with doctors and psychiatrists for early detection and treatment of Psychosis?
45. Is there anything else you want to add to the discussion?

Thank you very much for your cooperation.
